# Predicting speech fluency and naming abilities in aphasic patients

**DOI:** 10.3389/fnhum.2013.00831

**Published:** 2013-12-10

**Authors:** Jasmine Wang, Sarah Marchina, Andrea C. Norton, Catherine Y. Wan, Gottfried Schlaug

**Affiliations:** Neuroimaging and Stroke Recovery Laboratory, Department of Neurology, Beth Israel Deaconess Medical Center and Harvard Medical SchoolBoston, MA, USA

**Keywords:** aphasia, fluency, outcome, therapy, lesion size/volume, diffusion tensor imaging, functional MRI

## Abstract

There is a need to identify biomarkers that predict degree of chronic speech fluency/language impairment and potential for improvement after stroke. We previously showed that the Arcuate Fasciculus lesion load (AF-LL), a combined variable of lesion site and size, predicted speech fluency in patients with chronic aphasia. In the current study, we compared lesion loads of such a structural map (i.e., AF-LL) with those of a functional map [i.e., the functional gray matter lesion load (fGM-LL)] in their ability to predict speech fluency and naming performance in a large group of patients. The fGM map was constructed from functional brain images acquired during an overt speaking task in a group of healthy elderly controls. The AF map was reconstructed from high-resolution diffusion tensor images also from a group of healthy elderly controls. In addition to these two canonical maps, a combined AF-fGM map was derived from summing fGM and AF maps. Each canonical map was overlaid with individual lesion masks of 50 chronic aphasic patients with varying degrees of impairment in speech production and fluency to calculate a functional and structural lesion load value for each patient, and to regress these values with measures of speech fluency and naming. We found that both AF-LL and fGM-LL independently predicted speech fluency and naming ability; however, AF lesion load explained most of the variance for both measures. The combined AF-fGM lesion load did not have a higher predictability than either AF-LL or fGM-LL alone. Clustering and classification methods confirmed that AF lesion load was best at stratifying patients into severe and non-severe outcome groups with 96% accuracy for speech fluency and 90% accuracy for naming. An AF-LL of greater than 4 cc was the critical threshold that determined poor fluency and naming outcomes, and constitutes the severe outcome group. Thus, surrogate markers of impairments have the potential to predict outcomes and can be used as a stratifier in experimental studies.

## Introduction

Aphasia is a common symptom after left hemisphere stroke, and affected individuals often experience incomplete recovery despite receiving intense speech therapy after the acute stroke phase (Kertesz and McCabe, 1977; Wade et al., 1986; Pedersen et al., 1995; Engelter et al., 2006). Most natural recovery and traditional speech therapy-facilitated recovery from aphasia occurs during the first 6 months following a stroke (Nicholas et al., 1993; Moss and Nicholas, 2006; Lazar et al., 2010), although significant improvements in language functions have been described in case studies and in chronic patients undergoing intense and experimental therapies (Meinzer et al., 2005, 2007; Fridriksson et al., 2012; Zipse et al., 2012). Factors that can determine a patient's recovery from aphasia include lesion size and lesion site (Lazar and Antoniello, 2008; Marchina et al., 2011), as well as the initial level of impairment (Lazar et al., 2010). Other factors such as age, gender, degree of hemispheric language laterality, and small vessel ischemic lesion burden are also likely to play a role, but their significance in explaining some of the variance in outcome has not been well examined in larger-scale studies.

Voxel-based lesion symptom mapping has been used in the past to relate lesions to particular language behaviors in aphasic patients (Borovsky et al., 2007; Turken et al., 2008; Baldo et al., 2012; Magnusdottir et al., 2012). Our previous work took this approach one step further and related lesion volume to a speech- and language-relevant anatomical structure, creating a lesion load variable of the AF, which proved to be a superior predictor of speech production over lesion volume. Marcotte et al. (2012) also found that lesion volume *per se* was not a correlate of recovery in anomic patients. Introduced by Zhu et al. (2010), lesion load is a combined variable of lesion size and site that measures the effects of a lesion on easily definable and clinically relevant anatomical structures, such as white matter tracts derived from diffusion tensor imaging. The lesion load measure can serve both as a biomarker of speech fluency impairment and a predictor of aphasia outcome after a stroke. The method entails overlapping canonical probabilistic maps of a white matter tract (derived from diffusion tensor imaging) with patients' stroke lesion masks. One such speech-related tract is the arcuate fasciculus (AF), known from previous studies to play a critical role in the feedforward and feedback control of speech production (Breier et al., 2008; Hosomi et al., 2009; Saur et al., 2010b). The AF may have direct components (i.e., connections between temporal and inferior frontal brain regions) as well as indirect components (i.e., connections between temporal and parietal regions, and then parietal with frontal regions) (Catani et al., 2005). The horizontal portion of the AF mingles with the superior longitudinal fasciculus (SLF). Our understanding regarding the functional role of the AF in speech fluency/production and language functions in general is still evolving. It is thought that the AF is not only involved in auditory-motor mapping, including the feedforward and feedback control of speech-motor functions, but may also play a role in more domain general functions (Dick and Tremblay, 2012), such as syntactic processing, comprehension, and perception (Glasser and Rilling, 2008; Rolheiser et al., 2011).

On the other hand, contributions of the ventral white matter tracts (i.e., EMC and UF) in speech production remain unclear; despite fMRI and DTI evidence of the ventral stream's role in speech comprehension (Hickok and Poeppel, 2007; Saur et al., 2008, 2010b), lesion mapping, cortical and sub-cortical stimulation studies suggest that the ventral stream tracts (particularly the UF) do not play a dominant role in speech fluency and speech-motor functions (Duffau et al., 2009, 2013; Marchina et al., 2011; Moritz-Gasser and Duffau, 2013). Marchina et al. (2011) had explanded upon our original lesion load approach related to motor outcomes (Zhu et al., 2010) by overlaying lesions onto a canonical probabilistic map of the AF, the extreme capsule (EMC), and the uncinate fasciculus (UF) in 30 chronic patients with aphasia. They found that the Arcuate Fasciculus lesion load (AF-LL) best predicted speech fluency and naming, and that lesion loads of the EMC and UF tracts did not significantly correlate with measures of speech fluency and naming outcomes. In the present study, we re-examined possible contributions of the EMC and UF lesion loads using a larger group of patients; in addition, we updated the canonical maps of the AF, EMC, and UF tracts, which are now derived from probabilistic tracking in normal controls in lieu of deterministic tracking as used in Marchina et al. (2011).

Although the AF-LL has been shown to be a surrogate white matter marker of speech fluency after stroke (Marchina et al., 2011), speech production impairment and language recovery have also been related to the pattern of intact perilesional gray matter regions (Fridriksson, 2010; Fridriksson et al., 2012). In patients with relatively small left hemisphere lesions, particularly those sparing perisylvian regions of the temporal and inferior frontal cortices and allowing for reperfusion/recovery of those regions, recovery-related functional imaging changes are typically found in perilesional cortex (Heiss et al., 1997; Rosen et al., 2000; Crosson et al., 2007). Fridriksson et al. (2012) also found that the activation of perilesional areas within the language network was related to improvement in a naming task. While the contribution of contralesional homolog cortical activations toward recovery remains unclear (Heiss et al., 1999; Baumhauer et al., 2008; Meinzer et al., 2008; Bantis et al., 2010; Saur et al., 2010a,b; Schlaug et al., 2010), functional imaging studies in healthy controls suggest that both hemispheres are involved in the production and control of speech output when the rate of production is slow (Ozdemir et al., 2006). However, various studies have shown that speech functions are mostly left-lateralized (Knecht et al., 2000; Turken and Dronkers, 2011). For the current study, we defined a functional gray matter (fGM) map that included cortical brain regions active during speech production, and applied the lesion load method to this surrogate marker of lesion site and size. In addition, we tested prediction of a combined AF-fGM map, which was created from summing fGM and AF maps.

Since complex language function such as fluency, conversation, and naming are dependent on a cortical network of brain regions and connections through white matter tracts, it is clear that lesion map variables can only serve as surrogate marker for normal or impaired language function, and thus, do not allow us to draw firm conclusions that specific language functions are associated with particular structures. While a surrogate lesion marker may implicate the important role of a structure in the network of brain regions, it should not be assumed as the seed of the function.

The aim of the current study was to examine three surrogate biomarkers, a structural white matter lesion load (i.e., AF-LL), a functional gray matter lesion load (fGM-LL), and a combined structural and functional lesion load (AF-fGM-LL), in their ability to predict speech fluency and naming performance in a large group of chronic aphasic patients. In addition, we aimed to replicate the findings of our previous study comparing AF, EMC, and UF lesion load predictions of speech fluency and naming with updated probabilistic tracts. Lastly, we examined if we could identify a threshold of lesion load to differentiate severely affected patients from less severely affected patients using a receiver operation characteristic (ROC) approach.

## Materials and methods

### Patient group

The patient group comprised 50 chronic stroke patients [mean age: 55 (*SD*: 11), 10F, 40M] (Table 1); thirty of whom had been used in a previous study correlating AF-LL with measures of speech fluency (Marchina et al., 2011). All patients had some degree of non-fluent aphasia in the subacute stroke phase (according to a review of medical records), but showed varying degrees of recovery at their assessment timepoint (all patients were at least 6 months post-stroke with a median of 16 months post-stroke). Demographic data, language testing data, and lesion data are presented in Table 1. Patients with bi-hemispheric or brainstem infarcts, primary intracerebral hemorrhages, previous strokes identified either by MRI or medical record (besides the stroke that caused the aphasia), concomitant neurological diseases/disorders, and other aphasic syndromes such as pure anomia or global aphasia with severe reduction in speech output and severe comprehension deficits [defined as scoring less than 20% correct on Auditory Comprehension subtest scores of the Boston Diagnostic Aphasia Evaluation [BDAE] (Goodglass et al., 1983)], as well as significant cognitive impairments (less than the 50% correct on the Raven's Colored Progressive Matrices (RCPM) (Raven, 1995) were not included in this study. Our local Institutional Review Board approved this protocol and all subjects gave informed consent.

**Table 1:** **Patient Characteristics**.

**Biographical information**				**Ravens**	**BDAE Auditory Comprehension**			**Fluency/Naming measures**			**Lesion vol**	**Lesion loads**				
**Pt.#**	**Gender**	**Age @**	**Post-**	**(max = 36)**	**Word**	**Commands**	**Word**	**BNT**	**Words/**	**ClUs/**	**(cc)**	**AF-LL**	**fGM-LL**	**AF+fGM-LL**	**EMC-LL**	**UF-LL**
		**Ax**	**stroke**		**discrimination**		**repetition**		**min**	**min**		**(cc)**	**(cc)**	**(cc)**	**(cc)**	**(cc)**
		**(years)**	**(months)**		**(% correct)**	**(% correct)**	**(% correct)**	**(max = 15)**								
1	F	32	10	34	70.3	53.3	10.0	7	29.0	19.0	143.25	4.24	15.39	19.22	4.61	4.73
2	F	37	19	36	100.0	100.0	60.0	13	77.0	56.0	184.09	1.48	13.32	14.70	2.59	2.61
3	M	68	15	N/A	N/A	60.0	100.0	1	86.0	29.0	42.46	0.24	2.19	2.41	2.22	2.72
4	M	54	8	36	90.5	100.0	90.0	11	41.8	24.5	42.61	3.35	3.78	6.18	1.29	2.71
5	F	71	96	25	68.9	60.0	0.0	0	1.3	0.9	197.24	9.75	20.16	24.58	5.14	2.95
6	M	29	120	32	N/A	N/A	N/A	0	2.1	0.9	191.20	9.72	24.98	29.83	4.84	6.19
7	M	47	13	33	94.6	86.7	70.0	12	17.9	3.8	169.24	4.35	12.58	14.33	2.79	4.00
8	M	74	6	24	71.6	53.3	40.0	1	11.6	0.9	123.77	4.27	13.51	15.04	0.14	0.00
9	M	55	60	30	45.5	54.6	25.0	1	4.8	0.6	295.73	11.69	26.74	35.09	5.42	6.22
10	M	57	53	28	N/A	83.3	63.6	5	4.0	0.3	123.16	3.58	11.63	18.03	1.77	0.00
11	M	62	22	32	98 6	100.0	40.0	5	3.5	1.5	42.05	4.27	4.37	6.99	1.68	0.37
12	M	56	29	34	64.9	66 7	30.0	7	10.2	0.5	152.87	9.46	17.15	23.53	3.55	3.81
13	M	71	14	29	100.0	86.7	10.0	8	8.3	2.6	73.40	3.35	7.59	9.45	1.58	2.21
14	M	35	9	35	94.0	100.0	50.0	4	5.2	0.3	198.76	5.18	13.00	14.17	2.14	2.72
15	F	48	12	30	60.0	58.3	60.0	0	2.5	0.4	260.82	8.28	19.47	25.62	4.74	5.90
16	M	44	44	35	100.0	75.0	60.0	14	17.2	7.2	259.45	6.81	26.85	24.03	4.06	5.73
17	M	56	18	28	82.4	20.0	25.0	0	19.3	0.4	136.78	6.74	18.68	21.74	3.74	3.91
18	M	55	7	31	91.9	26.7	30.0	5	7.3	2.0	249.80	10.82	25.40	31.89	4.50	5.86
19	F	54	12	33	87.8	70.0	20.0	6	21.3	2.4	127.98	3.78	9.11	9.57	0.90	0.46
20	M	63	24	36	79.7	33.3	70.0	4	19.2	3.6	112.30	6.78	17.41	23.26	3.06	3.36
21	M	53	25	36	62.2	60.0	70.0	5	21.3	5.9	212.35	7.99	23.70	27.91	4.70	6.37
22	M	60	21	24	66.2	53.3	70.0	1	0.8	0.1	263.11	10.58	22.00	28.04	4.32	5.88
23	M	67	9	35	62.2	46.7	10.0	0	9.8	0.1	155.14	10.29	19.76	25.70	4.89	6.23
24	M	65	108	34	91.9	100.0	20.0	3	36.5	5.1	188.74	9.36	15.88	21.63	5.13	6.25
25	M	71	66	20	N/A	57.1	100.0	3	12.6	1.3	250.30	7.04	19.59	20.50	3.40	3.54
26	M	62	25	30	43.2	60.0	50.0	0	24.2	0.4	145.40	6.15	19.25	21.57	2.71	5.51
27	M	47	18	27	83.8	66.7	40.0	2	12.6	2.6	241.38	9.40	23.69	27.30	2.18	0.29
28	M	63	6	20	82.4	73.3	40.0	0	2.9	0.3	226.59	5.95	17.10	17.79	3.28	3.33
29	M	62	77	35	58.1	60.0	10.0	0	28.8	1.0	157.77	9.88	16.15	24.50	4.65	5.97
30	M	56	15	26	86.5	50.0	40.0	3	17.4	1.7	75.82	4.92	6.04	9.45	2.73	3.25
31	M	62	14	36	97.3	93.3	0.0	0	2.3	0.6	71.18	7.09	6.67	10.12	3.16	2.82
32	M	45	11	32	28.4	60.0	50.0	0	25.0	0.4	246.65	10.94	26.26	31.49	3.69	3.82
33	F	62	15	32	81.1	86.7	20.0	0	7.9	0.3	108.30	2.96	10.34	8.90	2.87	5.18
34	M	59	16	31	33.8	33.3	90.0	1	29.8	1.3	196.87	11.25	21.34	28.05	3.74	4.53
35	M	51	12	33	70.3	33.3	10.0	0	6.1	0.2	63.47	5.63	8.67	11.26	4.01	1.61
36	F	66	27	34	89.2	73.3	40.0	8	19.8	8.0	297.27	6.89	22.82	27.33	3.75	6.48
37	F	69	12	21	N/A	71.4	71.4	2	21.5	0.8	94.22	4.50	10.04	11.87	0.16	0.03
38	F	51	17	34	N/A	100.0	75.0	0	48.1	1.8	199.78	7.70	16.45	19.58	4.80	5.79
39	M	51	9	33	39.2	33.3	70.0	2	35.3	0.3	176.47	8.66	20.08	24.95	4.50	5.23
40	M	50	13	35	100.0	100.0	90.0	14	24.4	8.9	57.42	3.44	9.54	13.75	0.37	0.37
41	F	68	26	21	83.3	71.4	N/A	9	86.4	54.2	17.70	0.73	0.07	0.75	0.60	0.00
42	M	45	11	N/A	N/A	N/A	N/A	15	38.0	32.9	100.84	2.75	4.75	5.72	0.14	0 00
43	M	64	92	36	N/A	N/A	N/A	15	47.5	28.8	72.10	5.20	5.34	8.27	2.04	2 86
44	M	61	13	35	100.0	100.0	N/A	15	77.9	63.5	12.68	0.07	0.46	0.60	0.00	0.00
45	M	74	65	34	97.3	86.7	90.0	14	39.8	23.3	54.09	3.70	5.56	8.32	1.43	1.43
46	M	56	79	34	73.0	86.7	80.0	12	29.3	13.5	110.32	6.49	9.71	16.02	4.38	5.51
47	M	45	15	36	94.6	100.0	90.0	15	50.8	29.2	15.23	0.08	0.52	0.69	0.63	0.01
48	M	45	11	36	97.3	93.3	90.0	14	31.2	18.8	84.20	2.70	10.78	15.37	2.55	4.22
49	M	58	12	N/A	98.6	93.3	80.0	10	58.6	27.4	57.16	2.41	4.58	5.99	2.62	3.34
50	M	65	18	N/A	89.2	87.5	100.0	13	50.8	37.0	14.37	1.24	0.08	1.05	0.42	0.14

Table displays patient group's patient information (gender distribution, age at assessment (Age@Ax), time after stroke onset-to-assessment in months), Raven's score, BDAE Auditory Comprehension measures (expressed in % correct), lesion size (in cc), lesion loads (in cc), and fluency measures.

### Control group

Healthy subjects, age-matched with the patient group, were recruited in order to create canonical functional and structural maps. Functional MR images from one group of 12 healthy controls [mean age: 52 (*SD*: 13.9), 7M, 5F] were acquired during a speech production task and used to create canonical maps of activated gray matter (fGM). High-resolution Diffusion Tensor Images (DTI) from another group of age-matched 12 healthy controls [mean age: 58 (*SD*: 13.9), 8M, 4F] were used to create probabilistic, canonical maps of white matter tracts (AF, EMC, and UF) via probabilistic tracking. All healthy elderly control participants were right-handed, native speakers of English who scored within normal range in the Shipley/Hartford Verbal and Abstraction subtests (Shipley, 1940), which have been shown to be a predictor of IQ (Paulson and Lin, 1970). Our group of normal healthy control subjects was not tested on any fluency measures or naming tests. However, published data of a healthy control group suggests that the range of CIUs/min can be from 92 to 175 and the range for Words/min can be from 105 to 198 (Nicholas and Brookshire, 1993). Our group of patients, even the well-recovered patients, was well below those ranges (see Table 1).

### Behavioral measures

All patients underwent a battery of language tests to assess spontaneous speech production, naming, repetition, and comprehension, although the focus of this study was on speech and fluency measures. Conversational speech production was measured using the Correct Information Unit method (CIU) (Nicholas and Brookshire, 1993), and naming ability was assessed by the Boston Naming Test (BNT) (Goodglass and Kaplan, 1983).

In brief, speech fluency was assessed by transcribing videotaped conversational interviews comprising questions about biographical information (e.g., questions such as “where do you live, who do you live with?”), medical history (e.g., “what happened when you had your stroke?”), daily activities (e.g., “what do you usually do on Sundays?”), and descriptions of complex pictures [e.g., the Cookie Theft picture from the Boston Diagnostic Aphasia Examination and the picnic picture from the Western Aphasia Battery (Shewan and Kertesz, 1980) as well as similar pictures] with each patient. Transcriptions of patient's speech outputs were timed and coded by independent raters not involved in patient assessments. Our two main measures of speech fluency were words per minute (words/min), and correct information units per minute (CIUs/min). For the current study, we rescored all transcriptions, including those of the previous 30 subjects, in order to ensure consistency across all 50 subjects reported here. Words per minute is a common fluency measure, while CIUs/min is also referred to as “speech efficiency,” a measure that combines informativeness and fluency (Nicholas and Brookshire, 1993), and was found to have the highest correlations with the AF-LL in Marchina et al. (2011). In order to be counted as a CIU, words had to be intelligible, accurate, relevant, and informative to the prompt asked. To control for variation in length of responses, coders timed a full minute of patient speech production after each question or task description, and averaged the scores from each question/task description to produce a final overall score.

The BNT is a commonly used clinical assessment tool of naming ability in stroke patients. For this study, we used the 15-item Short Form published in the BNT 2nd edition (Kaplan et al., 2001). These 15 items correlated highly with the 60-item Standard Form (*R* > 0.9, *p* < 0.05). Other studies confirmed the 15-item Short Form to be an accurate assessment of naming (del Toro et al., 2011). Patients were not timed in their responses, and the maximum score was 15.

### Structural MR imaging

All stroke patients were scanned with a 3-Tesla General Electric MR scanner using a standard radiofrequency head-coil. T1-weighted MR images (voxel resolution 0.93 × 0.93 × 1.5 mm) were spatially normalized to the SPM T1-template (isotropic 2 mm voxel size) in SPM5 (Wellcome Trust Centre for Neuroimaging, London, UK) implemented in MATLAB (The Mathworks Inc., Natick, MA). Problematic normalizations were identified by visually inspection of registration, patients' T1-image normalizations were fixed by excluding the chronic ischemic lesion from the registration algorithm before normalization (Brett et al., 2001).

Twelve age-matched subjects [mean age: 58 (*SD*: 13.9), 8M, 4F] underwent diffusion tensor imaging (DTI) using a single-shot, spin-echo EPI sequence with the following parameters: *TR* = 10 s; *TE* = 86.9 ms; resolution 2.6 × 2.6 × 2.6 mm^3^; 30 non-collinear diffusion directions with a *b*-value of 1000 s/mm^2^ and 6 acquisitions with a value of 0 s/mm^2^. A total of 56 slices covered the entire brain including the brainstem. Postprocessing of DTI images and fiber tracking were done in FSL (www.fmrib.ox.ac.uk). Images underwent eddy current and head motion correction, and skull stripping with the brain-extraction tool (BET). Fiber probability distribution, diffusion tensor modeling, and fractional anisotropy (FA) images were generated during dtifit and bedpostx processing. The AF, EMC, and UF tracts were traced according to anatomical guidelines described in detail in Marchina et al. (2011).

### Reconstruction of the white matter tracts

**Figure 1:**
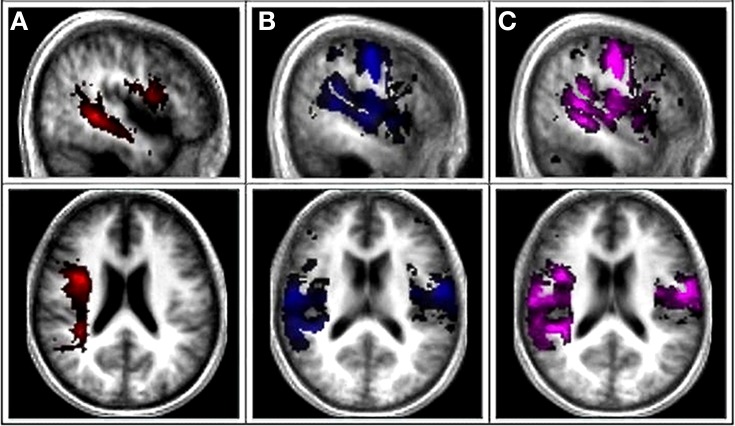
**Canonical AF, Functional gray matter (fGM), and Combined AF-fGM maps**. **(A)** First column is the canonical probabilistic map of AF tract derived from DTI overlaid onto a normalized averaged T1 brain in MRIcon. **(B)** Second column shows the canonical map of functional gray matter map. **(C)** Third column displays combined canonical map of combined AF-fGM map.

For the arcuate fasciculus (AF) tracts, we defined two regions of interest (ROIs) on the raw diffusion space FA maps in the white matter underlying the posterior middle temporal gyrus (approximately at *x* = −50 mm, *y* = −40, *z* = −4; MNI coordinates) and superior temporal gyrus (approximately at *x* = −50 mm, *y* = −40, *z* = 8; MNI coordinates). A third ROI was drawn on the same sagittal slice (approximately *x* = −50 mm, *y* = 14, *z* = 16) in the white matter underlying the pars opercularis of the posterior inferior frontal gyrus (IFG) as described in Marchina et al. (2011) (Figure 1). The AF was traced from the seed region in the IFG to the middle and superior temporal regions. Exclusion masks were drawn in the axial plane of the external capsule, in the coronal plane posterior to the temporal gyri, and in the sagittal plane of the region medial to the fiber bundle in order to exclude fiber projections that were not part of the AF.

For the EMC, a region of interest was drawn on a sagittal slice (*x* = −46, *y* = 30, *z* = 10) in the white matter underlying the pars orbitalis and triangularis in the IFG; a second region of interest was drawn on the same slice in the midportion of the white matter underlying the superior temporal gyrus (*x* = −46, *y* = −34, *z* = 8) (Marchina et al., 2011).

For the UF we drew coronal ROI in the anterior region of the corona radiata (*x* = −32, *y* = 38, *z* = 2), the anterior part of the temporal lobe where the UF adjoins the inferior fronto-occipital fasciculus, and in the white matter underlying the inferior (*x* = −34, *y* = 2, *z* = −8) and middle temporal (*x* = −34, *y* = 2, *z* = −24) gyri (Marchina et al., 2011).

All tracts were thresholded at 50th percentile to minimize extraneous fibers. The twelve resulting fiber tracts of each canonical map were normalized to the standard T1 MNI space in SPM5, then binarized and summed to create separate canonical probabilistic maps of the AF, EMC, and UF.

### Functional MR imaging

A separate group of twelve age-matched healthy control subjects [mean age: 52 (*SD*: 13.9), 7M, 5F] participated in functional magnetic resonance imaging that included performing an overt speaking task and a sparse temporal sampling fMRI design [for details of the overt speech task, see (Ozdemir et al., 2006)] implemented on a 3 Tesla GE scanner (BOLD sequence characteristics: *TR* 15 s, *TE* = 25 ms, voxel resolution = 3.75 × 3.75 × 5 mm^3^). The scanner task was synchronized with auditory stimuli via Presentation software (Neurobehavioral Systems, Albany, CA). The fMRI experiment consisted of 6 blocks of 20 task trials. Each block contained 15 overt speaking trials, and 5 control non-speaking trials. The auditory stimulus was recorded by a trained individual articulating 15 2-syllable phrases frequently used in everyday conversation (e.g., “goodbye,” “thank you”), as determined by The Dutch Center for Lexical Information (CELEX; http://www.mpi.nl/world/celex) (average frequency = 4658.8). The fMRI behavioral task was chosen to match the speech-motor and speech-fluency capabilities of our moderately to severely impaired stroke patients, and to reveal brain regions involved in speech motor functions. Subjects listened to an auditory cue, and then overtly repeated the exact phrase back at the same pace, or remained silent when there was no cue during control runs. Subjects' responses were recorded to verify proper adherence to condition. Auditory stimuli were presented in randomized order, and total scan time for each subject averaged 55 min.

### Functional image analysis

Functional scans were analyzed using SPM5 (Institute of Neurology, London, UK). The preprocessing steps included movement correction, spatial normalization to the SPM5 EPI template, and spatial smoothing with an isotropic Gaussian kernel of 8 mm.

The general linear model was used to estimate condition and subject effects; global differences in scan intensity were removed by scaling each scan in proportion to its intensity. A high-pass filter with 128 s cutoff setting was used to eliminate low-frequency drifts, and flexible finite impulse response measured the average BOLD response at post-stimulus time. Contrasts for speaking vs. silence for each subject were entered individually with a significance threshold of Family-wise Error at 0.05 (FWE).

### Canonical map creation

Functional Gray Matter (fGM) maps were extracted from the fMRI analysis, and multiplied with a standard gray matter mask from SPM5 anatomy toolbox to restrict the functional activations to gray matter. The FWE-thresholded maps of the twelve control subjects were then binarized and summed to create a canonical, probabilistic map of functional gray matter activation patterns (fGM) (Figure 1). Canonical structural white matter and functional gray matter (fGM) maps were summed to create the canonical probabilistic AF-fGM map (Figure 1).

### Lesion load calculation

To assess lesion damage to relevant functional and structural speech regions, we manually delineated lesion masks from the anatomical magnetic resonance images of the 50 stroke patients. One rater who was blind to subjects' behavioral outcomes manually drew patient lesion masks. The drawings were made using MRIcro software (http://www.mccauslandcenter.sc.edu/mricro/mricro/) on stroke patients' normalized T1-weighted images, with the coregistered FLAIR (0.5 × 0.5 × 5 mm^3^, 24 slices) images as a guide. No part of ventricular dilations or hemispheric atrophy that one can sometimes observe in chronic stroke patients was included in the lesion map. For verification, a second rater (also blind to patient behavioral scores) manually inspected and revised all lesion maps, and in addition drew lesion maps on a subset of patients. The inter-reliability for lesion map volume was >0.9. For the lesion load calculation, each stroke patient lesion map was individually overlaid onto the canonical AF map, canonical fGM map, and the combined AF-fGM map, as well as the EMC and UF maps to calculate the lesion load of each patient.

Lesion overlap calculations for each patient were done as described by Zhu et al. (2010). In short, the maps consisted of voxel intensities ranging from *I* = 0 (voxel is not present in any part of the tract or functional gray matter map in any subjects) to *I* = 12 (the voxel is present in the part of the tract in all subjects). The probability of each voxel being a part of the tract is 1/12 of that voxel's total intensity. Lesion load was calculated by summing the total intersecting voxels between the lesion map and the voxel intensity from each probabilistic map.

### Statistical analyses

All statistical analyses were completed with Predictive Analytics Software (PASW) SPSS (17.0.2). Linear and multiple regressions analyses were run with AF, fGM, and Combined AF-fGM lesion loads to predict behavioral measures of speech fluency and naming ability; age and stroke-to-assessment onsets were controlled for in each analysis. In addition, multiple regressions models were run to compare AF, EMC, and UF lesion loads in their ability to predict the behavioral outcome, while controlling for lesion size. Two outliers were excluded with residual analysis, where case-wise diagnostics showed those values were outliers at ±2.5 standard deviations.

Curve estimation analyses determined that the relationships between speech fluency outcomes and lesion loads were not represented well by linear trends. Given the volumetric nature of lesion load, we used a cube root transformation for linearity and to reduce variance (Woo et al., 1999; van den Elskamp et al., 2011). Naming ability was linearly related to lesion loads, so transformations were not applied for those regressions.

To assess if the biomarker *lesion load* can classify severe impairments of speech production, two-step cluster analyses were run to separate patients into severe and non-severe groups, using the behavioral measures words/minute, CIUs/minute, and naming (BNT). With automatic cluster detection, 2 groups for each variable were formed with a range of behavioral cutoffs between non-severe and severe groups. Discriminant analyses were run on the resulting groups to determine accuracy of the cluster cutoff. ROC curves identified the most accurate predictor of behavioral outcome among AF, fGM, and combined AF-fGM lesion loads and lesion volume, and defined the best threshold for stratifying severe/non-severe outcome.

## Results

All three lesion loads measures (AF, fGM, and combined AF-fGM) significantly predicted the two fluency and naming measures in linear regressions (Table 2), controlling for age and stroke-onset-to-assessment time. In multiple regression models, a comparison of AF-LL with EMC and UF lesion loads confirmed our previous finding that AF-LL was the only significant predictor of speech fluency and naming (Table 2); lesion volume was not significant in any multiple regression models relative to AF, EMC, and UF lesion loads (*p* > 0.05).

**Table 2:** **Linear regression coefficient values for AF, fGM, Combined AF-fGM, EMC, and UF lesion loads predicting CIUs/minute and BNT-Aphasia Short Form**.

		***R*^2^**	***p*-value**	**partial *R***
**A**				
CIUs/min (efficiency)	AF-LL^*^	0.66	0.000	−0.302^*^
	fGM-LL	0.58	0.000	−0.116
	AF-fGM-LL	0.59	0.000	
Words/min (rate)	AF-LL^*^	0.53	0.000	−0.321^*^
	fGM-LL	0.43	0.000	−0.044
	AF-fGM-LL	0.45	0.000	
BNT (naming)	AF-LL^*^	0.49	0.000	−0.249^*^
	fGM-LL	0.44	0.000	−0.102
	AF-fGM-LL	0.42	0.000	
**B**				
CIUs/min (efficiency)	AF-LL^*^	0.63	0.000	−0.547^*^
	EMC-LL	0.33	0.000	−0.042
	UF-LL	0.28	0.001	0.109
Words/min (rate)	AF-LL^*^	0.53	0.000	−0.529^*^
	EMC-LL	0.25	0.001	−0.03
	UF-LL	0.25	0.002	0.18
BNT (naming)	AF-LL^*^	0.44	0.000	−0.273^*^
	EMC-LL	0.35	0.000	−0.194
	UF-LL	0.28	0.001	0.197

Table 2A represents regression models with AF, fGM, and Combined lesion loads. Table 2B presents values from AF, EMC, and UF regression models. Semipartial correlation coefficients are shown from multiple regression models including both maps representing significant unique contribution to prediction models. Asterisks mark significant unique contribution to predicting behavioral outcome derived from partial R^2^ significance.

### Lesion load and speech fluency (CIUs/min)

**Figure 2:**
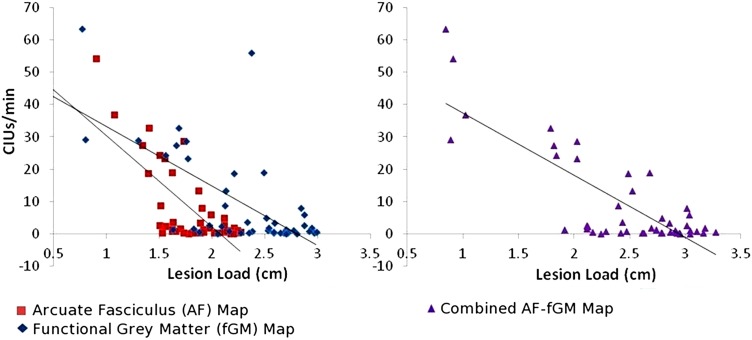
**CIUs/min vs. AF-, fGM-, and Combined AF-fGM lesion loads**. Lesion load is displayed on the *X*-axis, and CIUs/min is displayed on the *Y*-axis. AF-LL is shown in red, fGM lesion load is shown in blue, and combined AF-fGM-LL is shown in purple with corresponding regression curves. All regressions are significant (*p* < 0.01), and AF-LL significantly predicted better for speech efficiency through multiple regression analysis (*p* < 0.01).

In a multiple regression model, AF and fGM lesion loads significantly predicted the fluency measure CIUs/min (Adjusted *R*^2^ = 0.642, *p* < 0.01 for the overall model). However, AF-LL explained more variance in CIUs/min than the fGM-LL (AF-LL partial *R* = −0.30, *p* < 0.01; fGM-LL partial *R* = 0.12, *p* > 0.05). The combined AF-fGM-LL also significantly predicted CIUs/min (*R*^2^ = 0.59, *p* < 0.01), but did not predict more of the variance than either the individual AF or fGM lesion load models (Figure 2). In a separate analysis comparing AF-LL with EMC and UF-LL, while controlling for lesion size, AF was the only significant predictor of speech fluency (AF-LL partial *R* = −0.55, *p* < 0.05) compared to EMC and UF lesion loads (Table 2). Age and onsets-to-assessment were not significant predictors of fluency (*p* > 0.05).

### Lesion load and speech rate (words/min)

**Figure 3:**
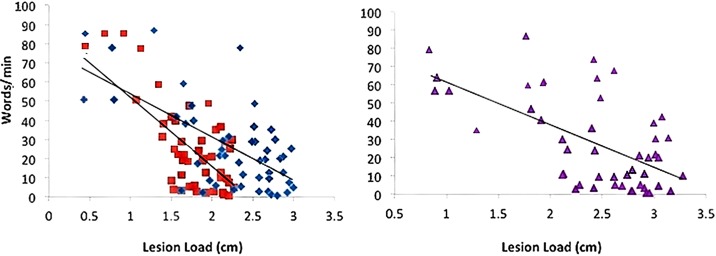
**Words/min vs. AF-, fGM-, and Combined AF-FGM Lesion Loads**. Lesion load is displayed on the *X*-axis, and Words/min is displayed on the *Y*-axis, and lesion loads are as labeled in Figure 1. All regressions are significant (*p* < 0.01), and AF-LL predicted words/min significantly better than fGM-LL (*p* < 0.05).

Words/min was predicted significantly in a multiple regression model by AF and fGM lesion loads (Adjusted *R*^2^ = 0.53, *p* < 0.01, for the overall model), as well as by the combined AF-fGM-LL (*R*^2^ = 0.45, *p* < 0.01). AF-LL predicted Words/min significantly better than fGM-LL (AF-LL partial *R* = −0.321, *p* < 0.05, fGM-LL partial *R*^2^ = −0.044, *p* > 0.05) (Figure 3). Controlling for lesion size, AF-LL was the only significant predictor of Words/min compared to EMC and UF lesion loads (AF partial *R* = −0.529, *p* < 0.05) (Table 2).

### Lesion load and naming ability (BNT)

**Figure 4:**
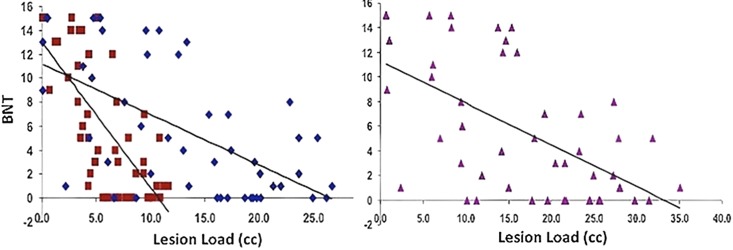
**Naming Ability vs. AF-, fGM-, and Combined AF-fGM Lesion Loads**. Lesion load is displayed on the *X*-axis, and naming ability is displayed on the *Y*-axis; lesion loads are as labeled in Figure 1. All regressions are significant (*p* < 0.01), and AF-LL significantly predicted better for naming ability through multiple regression analysis (*p* < 0.05).

Naming ability was predicted significantly in a multiple regression model with AF and fGM lesion loads (Adjusted *R*^2^ = 0.501, *p* < 0.01). Combined AF-fGM lesion load did not predict better than the individual maps (*R*^2^ = 0.418, *p* < 0.01). Naming was best predicted by AF-LL (partial *R* = −0.249, *p* < 0.05), and not by fGM-LL (partial *R* = −0.102, *p* > 0.05) (Figure 4). In the AF- EMC-, and UF-LL regression model, AF-LL remained the only significant predictor of naming (AF-LL partial *R* = −0.274, *p* < 0.05) (Table 2).

### Outcome group classification

A range of behavioral cutoffs for dividing severely and moderate-to-mildly affected subgroups was assessed by cluster analyses. Two-step cluster analyses with automatic grouping were run for behavioral classification of speech fluency into the two subgroups of severity, and the most accurate classification threshold was chosen for the behavioral cutoffs. A discriminant analysis confirmed a cutoff of 8–13 CIUs/min and 31–32 words/min to be 98% correct for dividing behavioral outcomes into severely and moderate-to-mildly affected groups (Figure 7). Lower and higher ranges of cutoffs in Words/min and CIUs/min were also tested (Table 3). For naming, automatic grouping in a two-step cluster analysis determined that those with a score of lower than 6 points out of 15 belonged to the severe impairment group, and a discriminant analysis confirmed this clustering was 100% accurate at classifying all data points; a range of naming cutoffs was also tested for cluster accuracy (Table 3).

**Table 3:** **Range of behavioral cutoffs for speech fluency and ROC prediction**.

**Behavioral measure**	**Cutoff range**	**Cluster classification accuracy (%)**	**AF-LL AUC ROC (%)**	**AF-LL threshold (cc)**
**CIUs/min**	8–13	97.9	93.5	3.74
	14–18	100	96.0	3.74
	19–21	100	95.3	3.39
**Words/min**	31–32	97.9	84.0	4.00
	33–35	100	81.9	3.74
	36–37	100	90.7	4.00
**BNT**	5	97.9	87.7	4.25
	6–7	100	90.0	4.01
	8	95.8	91.1	3.74

Table shows discriminant cluster classification accuracy of all data points, and resulting ROC prediction of AF-LL threshold and AUC. BNT measures are derived from the BNT aphasia Short Form, and maximum score is 15.

**Figure 5:**
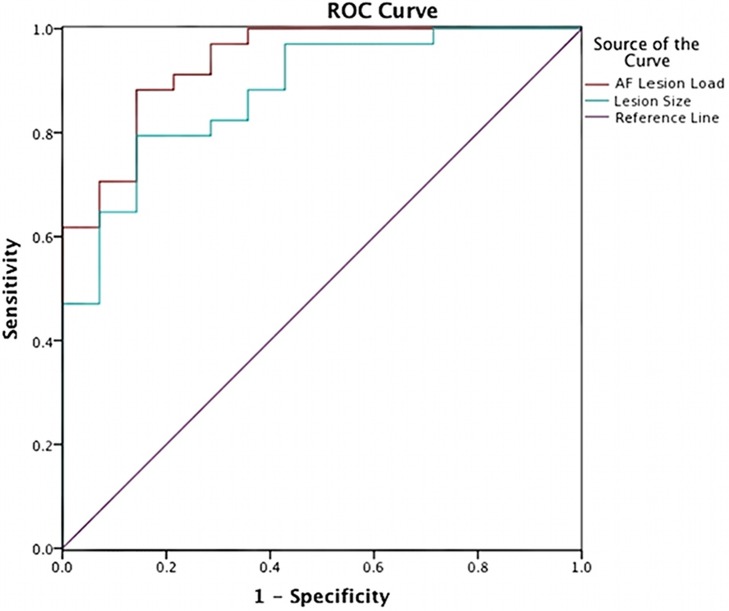
**Speech Fluency ROC curve shows prediction from AF-LL and lesion volume for speech fluency**. AF lesion load (in red) was the best at 96% in accuracy predicting severe and moderately/mildly affected groups at threshold at 3.75 cc.

**Figure 6:**
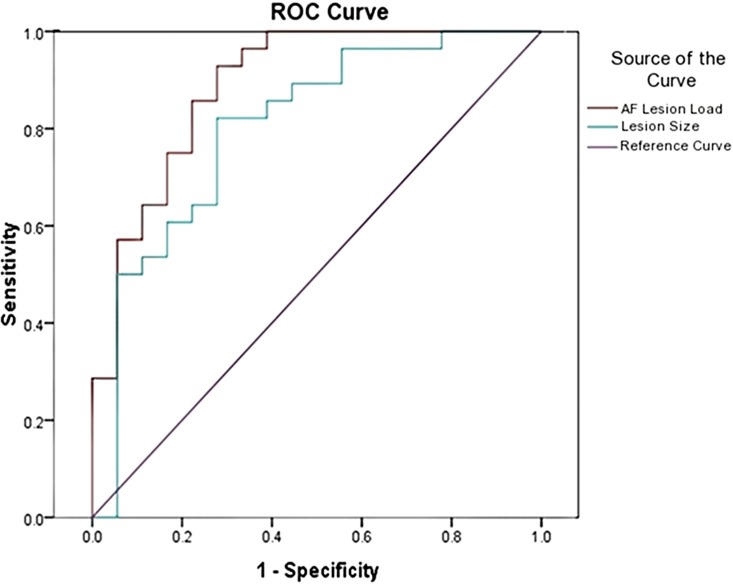
**Naming Ability ROC curve shows prediction from AF-LL and lesion volume for naming ability**. AF-LL was best at predicting naming with 90% accuracy, and a threshold for severe group at 4 cc.

Classification ROC curves were run for lesion loads and lesion volumes in order to determine the best lesion-load threshold for predicting severe and non-severe speech fluency and naming (Figure 4). With the previously determined behavioral cutoff at each range, AF-LL, fGM-LL, and combined AF-fGM-LL were all significant for predicting CIUs/min (*p* < 0.01) (data not shown). AF-LL was the best predictive model of severely impaired fluency (CIUs/min) (96% accuracy) with highest sensitivity (91%) and specificity (85%) (Figure 5) with the lesion load threshold for classifying a patient as belonging to the severe group around 3.75 cc of AF-LL (Table 3). Lesion volume was not as accurate a predictor as AF-LL with lower accuracy at 88, 80% sensitivity and 85% specificity, and a threshold for severe fluency at 105 cc lesion volume. For naming, AF-LL was again the best predictor with prediction accuracy at 90%, and a threshold for severely impaired naming classification at 4.01 cc of AF-LL with 91% sensitivity and 75% specificity, while lesion volume predicted naming with only 81% accuracy (Figure 6).

**Figure 7:**
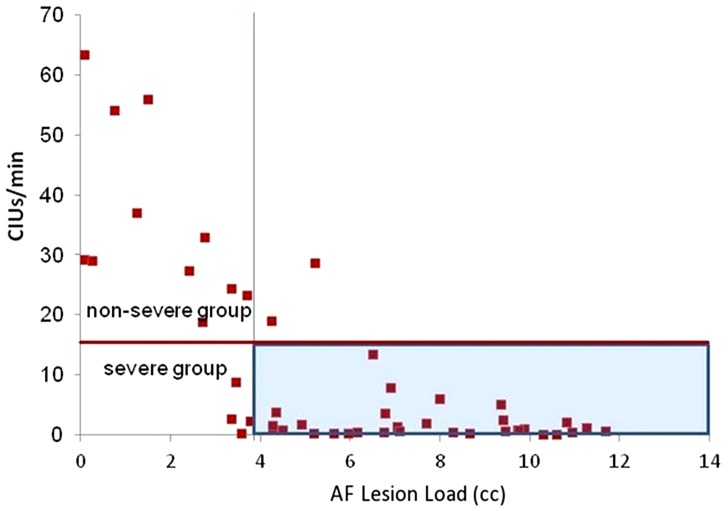
**Speech Fluency Classification of severe and moderately/mildly affected speech fluency outcome (shown by red horizontal line) at 15 CIUs/min**. The vertical line is at 3.75 cc of AF-LL determined by the ROC curve as the threshold for classifying severe outcome, which is highlighted in blue.

## Discussion

Similar to the findings in our previous study (Marchina et al., 2011), we found that AF-LL, in comparison to the novel fGM-LL and combined AF-fGM LL, best predicted our two measures of speech fluency (words/min and CIUs/min) and naming ability (BNT) in a large sample of patients. In addition, AF-LL provided the best classification of speech fluency and naming outcomes with >94 and 90% accuracy, respectively. An AF-LL threshold beyond ~4 cc classified a patient as belonging to the group with severe speech fluency and naming impairments.

The reason that the AF-LL emerged as the best predictor of impaired speech production may be due to its significant role in the feedforward and feedback control of speech production including naming and repetition (Damasio et al., 1996; Hickok and Poeppel, 2004; Borovsky et al., 2007; DeLeon et al., 2007; Tourville et al., 2008; van Oers et al., 2010). Previous studies have already reported that damage to the AF was predictive of speech repetition impairment (Fridriksson et al., 2009). These findings support AF-LL as a surrogate marker of the AF impairment. The AF also converges with the EMC on the lexical-semantic “hub” region of the middle temporal gyrus (Catani et al., 2005; Glasser and Rilling, 2008; Lawes et al., 2008; Turken and Dronkers, 2011) and has been associated with syntactic, semantic, and phonological tasks in language production and perception (Glasser and Rilling, 2008; Rolheiser et al., 2011). The involvement of the AF in many speech functions suggests that the degree of AF impairment in the left hemisphere may be a pivotal determinant of aphasia recovery (Rolheiser et al., 2011).

When we examined the lesion loads of the ventral stream represented by the EMC and UF tracts, despite EMC-LL and UF-LL providing modest predictions of speech fluency and naming outcomes, the AF-LL remained the most significant predictor in a multiple regression analysis. We also replicated our previous finding that lesion size was not a significant predictor relative to lesion loads, and our findings are consistent with those from Marchina et al. (2011); thus, we confirmed that ventral stream lesion loads, though significant independent correlates of naming and fluency, do not provide the best predictions relative to AF lesion load. These results support an emerging theory that the relationship between dorsal and ventral streams in speech are not easily separated by localized speech functions, and could indeed have a synergetic relationship as proposed by Rolheiser et al. (2011).

In the current study, we also replicated results from Marchina et al. (2011) and those of Marcotte et al. (2012) with regard to lesion volume and its marginal predictive ability of outcome and recovery. Although lesion volume independently predicted speech outcomes, it does not survive significance in a multiple regression model with AF-LL. This may be because lesion volume significance was derived from the damage to relevant language brain structures such as the AF, and does not contribute unique prediction to speech outcome. In aphasia research, there are differences in methods for determining lesion size/location, stroke type, and behavioral tasks from study to study, so it is difficult to define a strict lesion cutoff that determines outcome. To our knowledge, no group has yet established a clear cut-off value for lesion volume that predicts speech outcome; however, our AF-LL variable may have the potential to provide such a value (e.g., an AF-LL of 4 cc or more seems to be associated with severe non-fluent aphasia). This would obviously have to be replicated and further tested in subsequent studies.

Even though the AF-LL is the best predictor of speech fluency among the three white matter tracts examined, a functionally defined gray matter template could have been possibly more predictive of speech fluency impairment. Our rationale for choosing a functional gray matter map was based on previous studies showing that variations in perilesional activations are related to recovery from aphasia (Meinzer et al., 2008; Fridriksson, 2010; Fridriksson et al., 2010; Saur et al., 2010a; Hamilton et al., 2011). Although Saur et al. (2010a) combined fMRI with Diffusion-Weighted-Imaging (DWI)-derived lesional data, they did not find an improvement in their outcome predictions (Saur et al., 2010a). We assume that the lesion load variable that we used and combined with the fGM maps was more specific to the interconnected functional regions that were damaged in our sample of subjects. Furthermore, we found the lesion load of our fGM map to be correlated with speech fluency and naming abilities after stroke, but it did not explain as much of the variance as AF-LL. This difference could be due to the smaller size of the structural canonical map, which connects the core regions of the speech-motor network. In contrast, the fGM map encompassed the wider and more diffuse functional network necessary for word/phrase repetition, which may have included cortical regions beyond the critical core regions of the speech-motor network.

While there were various options of fMRI tasks that could have been used to define the fGM map, the word/phrase repetition fMRI task used in the current study allowed us to exert a high level of control on the timing and duration of the speech production task, which is important in sparse temporal fMRI designs. Furthermore, the resulting pattern of activation revealed a speech motor network that included regions in the premotor, SMA, inferior frontal, primary inferior sensorimotor, and posterior superior temporal regions, and is similar to speech production activation patterns reported in other publications (Saur and Hartwigsen, 2012). Lastly, our choice to use a word/phrase repetition task was also driven by some overarching designs that were not necessarily directly related to the analysis in this particular study, but had to do with several ongoing studies examining fMRI networks in age matched normal controls and aphasic patients. Thus, in order to capture some degree of speech production in all participants—even the most severely impaired patients—a strictly controlled word/phrase repetition fMRI task was in our opinion the best option in comparison to other fMRI tasks such as conversation or picture naming that included additional confounds e.g., untimed responses and/or use of visual stimuli. Despite the limitations of finding a suitable fMRI task for a wide variety of healthy age-matched controls and patients with various impairments, the fGM-LL was still a robust predictor for our speech fluency and naming outcomes, indicating that this method is promising for future investigation.

Although both AF-LL and fGM-LL predicted speech production individually, the lesion load of the combined AF and fGM maps did not provide a significantly better prediction than either of the variables alone. Although other studies have combined DTI and fMRI techniques to confirm functional connectivity between activated speech regions (Saur et al., 2010b), to the best of our knowledge our current study is the only one using a combined cross-modality model that included DTI, fMRI, and lesion load information for predicting aphasia outcome.

For predicting severe fluency and naming outcomes, the ROC classification model indicated that once AF-LL exceeds ~4 cc threshold, conversational fluency and naming in the outcome group are severely impaired; this threshold remained consistent through a range of behavioral cutoffs. Although other studies have correlated lesion size with speech outcome (Kertesz et al., 1979; Naeser et al., 1981), to our knowledge no other study has used a lesion-load threshold of functionally relevant gray or white matter to classify the severity of speech fluency impairment. This threshold of 4 cc AF-LL could be very useful as a clinical predictor of outcome, especially since patients in the severely impaired group could adopt alternative and intensive therapies in order to retrain or involve right hemisphere speech-motor networks, such as Melodic Intonation Therapy or non-invasive brain stimulation applied to the right hemisphere (Schlaug et al., 2008, 2010, 2011), or for the less AF-impaired group (i.e., those with small left AF-LL) to focus on rehabilitating the ventral stream or supporting perilesional neural networks of speech and language function with or without non-invasive brain-stimulation. Albeit our model is relatively simple, the clustering method provides an objective grouping of the behavioral outcome, while AF-LL seems highly accurate for stratifying non-fluent aphasic stroke patients in the chronic stage, especially compared to overall lesion size.

Although there are other options that may have been considered appropriate behavioral measures for determining degree of speech-motor impairment and/or degree of improvement in post-stroke aphasia, we chose CIUs/min (Nicholas and Brookshire, 1993) and Words/min as measures of speech fluency, because each measure provided important information regarding a patient's impairment. Words/min revealed patients' articulatory agility, but lacked “informativeness” (accuracy of information) or efficiency of the speech; CIUs/min was designed to be an accurate, quantitative measure of functional speech, and served to quantify both informativeness and efficiency of the patients' speech output. However, without Words/min, CIUs/min does not always reveal the full nature of the impairment. Using both measures has allowed us to capture multiple aspects of deficits and improvements in the speech output of non-fluent aphasic patients with relatively wide range of impairments.

A few caveats apply to our findings. Although our model reveals a strong relationship between left AF-lesion load and patient outcomes on measures of fluency and naming, we could not take into account remote effects of lesions onto non-lesional brain regions contributing to the behavioral phenotype due to a disconnection (Weiller et al., 1993), or the variable size of the right AF and homolog/homotop speech regions on the right hemisphere as showing plastic changes post-stroke and over time (Rosen et al., 2000; Crinion and Price, 2005; Saur et al., 2006; Raboyeau et al., 2008; Schlaug et al., 2009). Secondly, since the current study was partially a replication study including 30 original patients from a previous study out of 50 presented here, the results could possibly be biased; however, the updated probabilistic canonical white matter tracts and the functional GM maps as well as the combined structural and functional maps to determine lesion loads were new for all patients; thus, besides a significantly larger patient population, our investigation has novel aspects that go beyond a simple replication of a previous study. Thirdly, the white matter tracts were reconstructed in age-matched healthy elderly controls using 30 diffusion directions. There is some debate in the literature with regard to the optimal number of diffusion directions to be used. While some argue that a higher number is better, others have argued that 30 might be adequate (Mukherjee et al., 2008). There is no accepted standard on the optimal number of diffusion directions, although multicenter reliability studies have used 30 directions recently and found acceptable variations across sites (Magnotta et al., 2012). Nevertheless, the number of DTI directions may not be as problematic for our study, since we have aggregated white matter tracts from healthy, matched elderly controls into a probabilistic canonical map for the purpose of calculating a lesion overlay, rather than focusing on an examination that would require more directions for optimal DTI acquisition (e.g., fiber integrity in or surrounding an ischemic lesion in patients). Lastly, the generalization of our predictive model may be limited, since we exclusively recruited a group of patients with speech fluency impairments who were mainly classified as non-fluent aphasics in the acute stroke phase. Furthermore, it is possible that recovery from aphasia can continue to occur in the chronic stage, and thus correlations between lesion markers and behavioral profiles could change over time and our predictions could show some dependency on time after stroke. Our current set of data does not necessarily support this, but larger numbers of patients would need to be tested to examine this in more detail. Our model of outcome predictions using the AF-LL can be tested in acute, subacute, and chronic stroke patients with a wider range of aphasia classifications.

Whereas the importance of the AF-LL as a biomarker for degree of impairment in both speech fluency and naming ability in chronic stroke patients was established by our earlier publication (Marchina et al., 2011), the present study both confirms the original findings in a larger patient sample, and compares the predictability of AF-LL to that of a new measure—fGM-LL. Furthermore, the AF-LL marker can help stratify patients by their level of impairment (e.g., mild/moderate and severe), which should improve outcome predictions and thus, help (1) identify those who are likely to benefit from particular interventions and/or experimental treatment studies, (2) guide clinicians in the selection and implementation of such treatments, and (3) maximize treatment time with the goal of improving upon predicted outcomes. Two major advantages of the AF-LL marker are its simplicity and practicality, since there is no need for additional high-resolution MR imaging beyond what is typically acquired at the time of stroke onset. Furthermore, this measure can easily be calculated and used in both research and clinical settings, making this potentially valuable tool more widely available to stroke professionals. Although we see great potential for such a neuroimaging biomarker, the predictability of AF-LL should also be compared to other behavioral measures and combination of imaging and behavioral measures in future studies. Furthermore, future studies will be needed both to test and refine the AF-LL as a surrogate marker of speech fluency, and more deeply investigate its value in longitudinal outcome studies of stroke survivors with aphasia.

### Conflict of interest statement

The authors declare that the research was conducted in the absence of any commercial or financial relationships that could be construed as a potential conflict of interest.
